# The hidden battlefield: platelet function in the wake of severe trauma

**DOI:** 10.3389/fimmu.2026.1830721

**Published:** 2026-07-03

**Authors:** Zuyang Wu, Fang Li, Qiang Chen, Zhijia Kang, Shengjun Cao, Quan Li

**Affiliations:** Department of Burn Surgery, The Third Affiliated Hospital of Inner Mongolia Medical University (Inner Mongolia Baogang Hospital), Institute of Burn Research of Inner Mongolia, Baotou, China

**Keywords:** burns, coagulopathy, hemorrhagic shock, inflammation, platelet, traumatic brain injury

## Abstract

Trauma contributes significantly to the global burden of suffering and death, and interrupts the complex balance between hemostasis and the immune system; platelets are key in integrating hemostatic and immune functions. This review will provide an overview of how different types of traumatic injury including traumatic brain injury, hemorrhagic shock, and burns impact platelet function through both separate and interconnected mechanistic pathways. Moreover, this review will examine in detail the multiple manifestations of trauma-induced platelet dysfunction, including but not limited to impairments in platelet activation, adhesion, aggregation and degregulation of granule secretion patterns. In addition, this review will describe how these platelet defects alter the dual roles of platelets as regulators of immunothrombosis and systemic inflammation. At a cellular level, separate types of trauma converge on shared molecular signaling pathways while utilizing different pathophysiological pathways to produce a complex network of regulations that control how platelets function. The purpose of this review is to present a comprehensive synthesis of current literature to provide a more complete overview of the mechanisms involved with trauma-related platelet abnormalities and to provide a framework for developing platelet-targeted therapies in the future.

## Graphical Abstract

**Figure:**
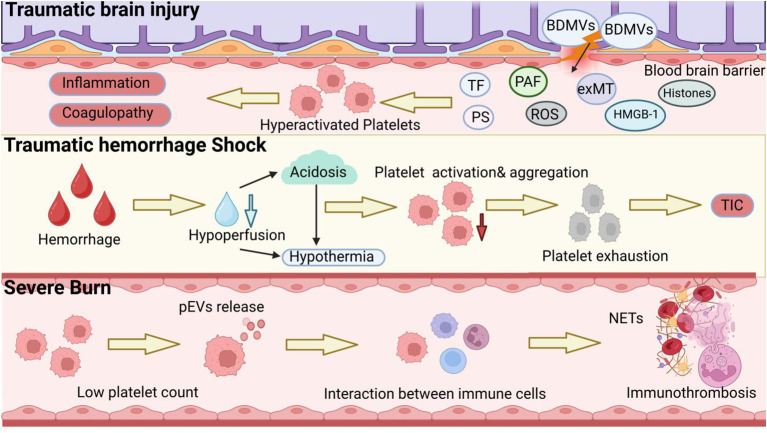

## 1 Methods

Our study adopted a narrative review design. We first defined the topic of our literature, focusing on the roles of platelet in coagulation and inflammation in different contexts of trauma. During initial literature search, we employed combinations of keywords such as platelet/trauma, platelet/inflammation, and platelet/traumatic brain injury, among others. The retrieved articles were then screened based on their titles and abstracts. Selected articles were subsequently examined in full detail, which allowed us to identify emerging topics and refine our search strategy. After that, we adopted a targeted approach focused on specific terms that we found in those articles, such as platelet-derived extracellular vesicles and neutrophil extracellular traps. By coupling these terms with the original keywords (e.g., platelet-derived extracellular vesicles/trauma), we retrieved additional relevant literature and ultimately yielded the final content of citations presented in this work.

## 2 Introduction

Trauma remains a leading cause of mortality and disability, impacting patients’ quality of life and presenting significant public health issues (1). Uncontrolled hemorrhage represents one of the most common causes of traumatic death and occurs frequently in combination with coagulopathy due to the impact of trauma on hemostasis (2). Of trauma patients who have an increased procoagulant state, around one quarter display trauma induced coagulopathy (TIC), and these patients are associated with higher mortality (1–3). A major determinant of outcomes in critically injured patients is the complex and dynamic nature of hemostatic function, which becomes significantly dysregulated following severe trauma (4). Platelet serves as a critical element of hemostatic and inflammatory function; this component has recently received increased attention because of its diverse roles — not only in hemostasis, but also in inflammation and immunity, as well as in the repair or regeneration of damaged tissues (5, 6).

Trauma includes many different and complex pathological processes (7, 8), which disrupt platelet function to influence physiological homeostasis and vascular circulation (9). Each of these processes can lead to significant complications and even death. For example, traumatic brain injury (TBI) is characterized by localized hemorrhage within the confined space of the cranium. Even minor bleeding can lead to severe neurological injury as a result of increased intracranial pressure and subsequent ischemic injury (10). On the other hand, traumatic hemorrhagic shock (THS) occurs when there is an excessive and uncontrolled loss of blood due to rupture of major blood vessels or organ damage. This can lead to systemic hypoperfusion, hypothermia and metabolic acidosis (11). Severe burns also produce widespread inflammatory reactions in the exposed skin, which cause large amounts of fluid to leak from the circulatory system, resulting in severe fluid shifts, increased capillary permeability, and a hypermetabolic state (12). The way in which an individual presents with coagulopathy after suffering trauma will vary considerably depending on the type of injury suffered. Localized hyperfibrinolysis and platelet dysfunction can often be observed with traumatic brain injury, while hemorrhagic shock usually presents with consumptive coagulopathy and dilutional thrombocytopenia. The sequence of events associated with burns is platelet hyperactivation, followed by exhaustion and suppression of bone marrow production.

Therefore, platelets play a variety of roles in all three conditions, affecting hemostasis, the utilization of pro- and anti-inflammatory mediators, and the immunomodulation of interactions between leukocytes and endothelial cells (6, 13). This review will methodically explore how each cause of traumatic injury, such as TBI, THS and severe burns cause a specific effect on platelet function and analyze the central role of platelets in coagulation and inflammation from the perspective of trauma and attempt to translate this understanding into clinically applicable strategies (9).

### 2.1 Platelet function alterations after Traumatic brain injury

In TBI, platelets exhibit functional changes in response to a microenvironment of the Central Nervous System (CNS) characterized by damage-associated molecular patterns (DAMPs) and unique brain patterns. Inflammatory changes resulting from those neuroendothelial environments alter platelet activation and aggregation, which can ultimately lead to coagulopathy. TBI-related coagulopathy differs from TIC since it is activated via specific neurohumoral pathways and can occur even with mild TBI, without extensive tissue loss or systemic hypoperfusion (14, 15). Acute coagulopathy can occur equally in isolated TBI as well as those with associated extracranial trauma, regardless of the critical causal factors typically present in coagulopathy related to extracranial traumatic injury, including massive tissue injury and/or significant blood loss.

Following TBI, there is an initial hypercoagulable phase and an increased propensity of platelets to aggregate, along with an effective cessation of several fibrinolytic systems that serve to dissolve aggregated platelets (17). In this phase, platelets become hyperactive and can be activated through diverse pathways to exert procoagulant and proinflammatory responses with the release of brain-derived procoagulant microparticles and DAMPs (14, 15). Some patients will develop coagulopathic disorders in the presence of normal platelet count, suggesting that platelet hypo-function can occur independent of platelet numbers. As the TBI progresses to the late stage of the injury, the hypocoagulable state reappears due to platelets losing their ability to aggregate, a phenomenon also referred to as platelet exhaustion (15–17). While the exact mechanism(s) for these dysfunctions are still being studied, multiple studies have demonstrated that the major manifestations of platelet dysfunction involve changes in the ability of platelets to respond to known agonists like ADP, collagen, thrombin, arachidonic acid (AA) and to aggregate. Without adequate hemostasis resulting from platelet hypo-function, the likelihood of experiencing bleeding and/or microvascular thrombi after TBI is considerably increased (18). Alternatively, based on experimental data in several murine head injury models, it is indicated that platelet dysfunction may arise from the cascading event of platelet hyperstimulation (19) or the release of platelet-derived extracellular vesicles (pEVs) that inhibit receptor expression (20, 21). Additionally, in a blood study collected by trauma patients, we can infer that inadequate platelet aggregation occurs due to consumption of von Willebrand factor (vWf) following an endothelial insult (22).

#### 2.1.1 Blood brain barrier disruption

The BBB is a complex structure composed of brain microvascular endothelial cells, astrocytes, pericytes, microglia, and neurons, as well as the surrounding basement membrane which plays a critical role in protecting neural tissue from damage and is a major contributor to the pathophysiology of many neurological disorders (18). The disruption of the BBB allows brain-derived cellular debris to enter the peripheral circulation, thereby activating platelets. The activated platelets release vesicles that promote coagulation and inflammation, leading to systemic coagulopathy and an inflammatory response. This pathological cycle not only worsens the brain injury but also creates a pathway to extensive systemic complications such as DIC and organ failure, demonstrating that the effects of what seems to be a localized cerebral injury are far-reaching (18, 23).The mechanical injury disrupts the microstructure to enable leukocytes to migrate into damaged tissue, triggering ischemic/inflammatory cascade continues to damage and leaky the BBB (24). Following infiltration, leukocytes initiate microglial activation, contribute to axonal damage, and promotes inflammation by releasing cytokines and chemokines that recruit more neutrophils and monocytes, thereby further amplifying tissue damage and sustaining the inflammatory response (25).

#### 2.1.2 BDMVs alter platelet behaviors

Brain-derived microvesicles (BDMVs) are shed from neurons, astrocytes, and endothelial cells. After TBI, BDMVs are critical to the development of a hypercoagulable state and the rapid transition to consumptive coagulopathy (23, 26, 27). BDMVs contain various procoagulant factors, such as tissue factor (TF), phosphatidylserine (PS), platelet-activating factor (PAF), and extracellular mitochondria (exMt), that induce platelet activation and enhance coagulation through distinct pathways (28). Both murine models and patients’ data have demonstrated that BDMVs can enter the circulation rapidly post-TBI, establishing a hypercoagulable state (14, 23). Laboratory studies have shown that BDMVs can affect platelet function both directly and indirectly, hindering platelet adhesion and aggregation and transferring bioactive material to alter endothelial cell behavior (29).

BDMVs engage with platelet receptors such as glycoprotein VI (GPVI) and integrin αIIbβ3, thereby promoting adhesion, aggregation, and thrombus formation. Besides, they facilitate intercellular communication between platelets and leukocytes, leading to aggregate formation that exacerbates vascular inflammation and microvascular occlusion. This process contributes to secondary brain injury in TBI.

##### 2.1.2.1 TF and PS and coagulation cascade in TBI

BDMVs containing TF potentiate coagulation processes and interact with platelets, enhancing their adhesion, aggregation, and the release of their contents (14, 23). TF, typically sequestered within brain tissue, enters the circulatory system post-injury, either independently or associated with BDMVs, and initiates the extrinsic coagulation pathway by activating factor X, thereby promoting thrombin and fibrin generation (16). PS contributes to the formation of complexes that accelerate the production of thrombin and fibrin. Taken together, some findings through mathematical modeling and *in vitro* experiments demonstrate that TF-initiated thrombin generation is critical to the pathophysiology of both platelet-driven coagulopathy and neuroinflammatory processes following TBI (30, 31). Thrombin, generated through TF activation, augments platelet reactivity and neuroinflammation by activating protease-activated receptors (PAR-1 and PAR-4) on platelets, leading to calcium mobilization and cytoskeletal alterations. In addition, thrombin can induce CCR2-dependent leukocyte recruitment and amplify inflammation via protease activated receptors (30). In adult male mice subjected to fluid percussion injury, the interaction between platelets and microparticles facilitates microvascular thrombosis, thereby intensifying the procoagulant state and perpetuating a thromboinflammatory cycle (26).

##### 2.1.2.2 PAF

PAF is synthesized by neurons, glial cells, and cerebrovascular endothelial cells, and is abundantly present in the brain, spinal cord, and other central nervous system tissues (14, 32). The actions of PAF on target cells are primarily mediated through PAF receptor, a specific G-protein-coupled receptor expressed on various cell types, including platelets, endothelial cells, microglia, and neurons. Besides that, requiring potassium efflux and calcium influx, PAF can also activate inflammasome like NLRP3, which seems to be associated with the pathogenesis of multiple noncommunicable inflammatory, metabolic, and neural diseases. More interestingly, although PAFR is the well-known receptor for PAF, its biofunction of inflammasome activation is regarded independent of PAFR (33). Following TBI, pathological conditions lead to a substantial increase in PAF production by remodeling membrane glycerophospholipids via phospholipase A2 and acetyltransferase (32). PAF is released into the tissues and activates multiple autocrine and paracrine signaling pathways that significantly maintain homeostasis in neurovascular function (33, 34). The activation of phospholipase C and phospholipase A2 by PAF occurs in platelets, leukocytes, and endothelial cells, leading to the hydrolysis of phosphoinositides, the activation of intracellular calcium, and the release of arachidonic acid, a platelet agonist from cellular membranes. Collectively, through both PAF and eicosanoids, secretion of dense and α-granule contents, and sustained platelet aggregation are promoted, thereby increasing the risk of occlusive microthrombus formation in the injured cerebral microcirculation. Collectively, these PAF-mediated signaling pathways contribute to thromboinflammatory cascades that link CNS injury with subsequent microvascular dysfunction, coagulopathy and progressive neurodegeneration (33). Last but not least, PAF also involves the breakdown of the BBB to release more of PAF, TF and other procoagulant microvesicles (14).

##### 2.1.2.3 exMTs

Following TBI, a substantial number of circulating BDMVs in the bloodstream are exMTs released from damaged neurons, glia, and endothelial cells (32). These exMTs retain metabolic functions, including membrane potential and electron-transport capacity, enabling them to consume oxygen and produce reactive oxygen species (ROS), such as superoxide and hydrogen peroxide. On the one hand, these ROS may activate nearby platelets and make platelets highly procoagulant, triggering signaling cascades and oxidizing membranes (35–37). This occurs because of the oxidative modification of GPIbα on platelets, which increases GPIbα’s ability to bind to vWF, leading to platelet adhesion and aggregation; these are significant processes needed for creating microthrombi in the cerebral microvasculature. Besides, platelets exposed to long-term oxidative stress undergo apoptosis and produce procoagulant microparticles (38–40). On the other hand, exMTs may aggravate neuroinflammation through inducing the polarization of microglial M1-type pro-inflammatory phenotype (30). Platelet function after TBI is mediated by ROS in addition to their role as cell killing agents.

#### 2.1.3 The release of DAMPs in TBI

TBI leads to cell death and stress, resulting in the large release of endogenous DAMPs, including ATP, High-mobility group box 1 (HMGB-1), mitochondrial DNA, histones, nucleosomes, and S100 proteins, as well as inflammatory cytokines (IL-6, TNF-α). These are all important mediators of how platelets become primed to respond to low-potency agonists such as ADP and collagen by upregulating surface receptors and increasing intracellular calcium flux,which may potentially link thrombosis with inflammation. The sources of DAMPs are so diverse that they include damaged or surrounding brain cells, migrated leukocytes and even platelet itself (7).

Among these DAMPs, the evaluated level of HMGB-1 and histones can be easily observed in severe trauma patients (46). HMGB-1 is a nuclear protein and is released into the extracellular environment through necrotic or damaged neurons or actively secreted by immune cells such as macrophages and microglia (41, 42). When HMGB-1 binds to and activates TLR4 on platelets, it triggers additional proinflammatory cytokine release, especially interleukin-1 (IL-1), and provides a source of HMGB-1, exacerbating neuroinflammation by recruiting and activating other immune cells in the CNS (43). Moreover, recent data indicate that HMGB-1 is critical in promoting inflammation and tissue injury (44). The current evidence indicates that HMGB-1 induces systemic immune activation and inflammation in TBI by being released from damaged neurons and activated immune cells. In this regard, platelets are important in modulating the body’s inflammatory response by releasing HMGB-1 from their granules, thereby amplifying localized neuroinflammation following TBI. Histones are regarded as binding agents for genetic materials. Released from damaged tissue, histones serve as DAMPs and possess procoagulant and proinflammatory effects (45). Histones mainly consist of H1, H2A, H2B, H3, and H4, among which H3 and H4 play a predominant role in their effects on platelets (46). Histones bind to Toll-like receptors (TLRs) on platelets, thereby producing thrombin and triggering the coagulation cascade. Multiple animal *in vitro* experiments have confirmed the promoting effect of histones on platelet activation and aggregation, through the NF-κB pathway (47), calcium influx and recruitment of plasma adhesion proteins (48), as well as upregulation of P-selectin (49). Besides the interactions of histones with platelets, histones can be released from neutrophils to stimulate coagulation (48). In addition, histones can activate leukocytes through TLRs and exacerbate endothelial injury.

**Figure 1:**
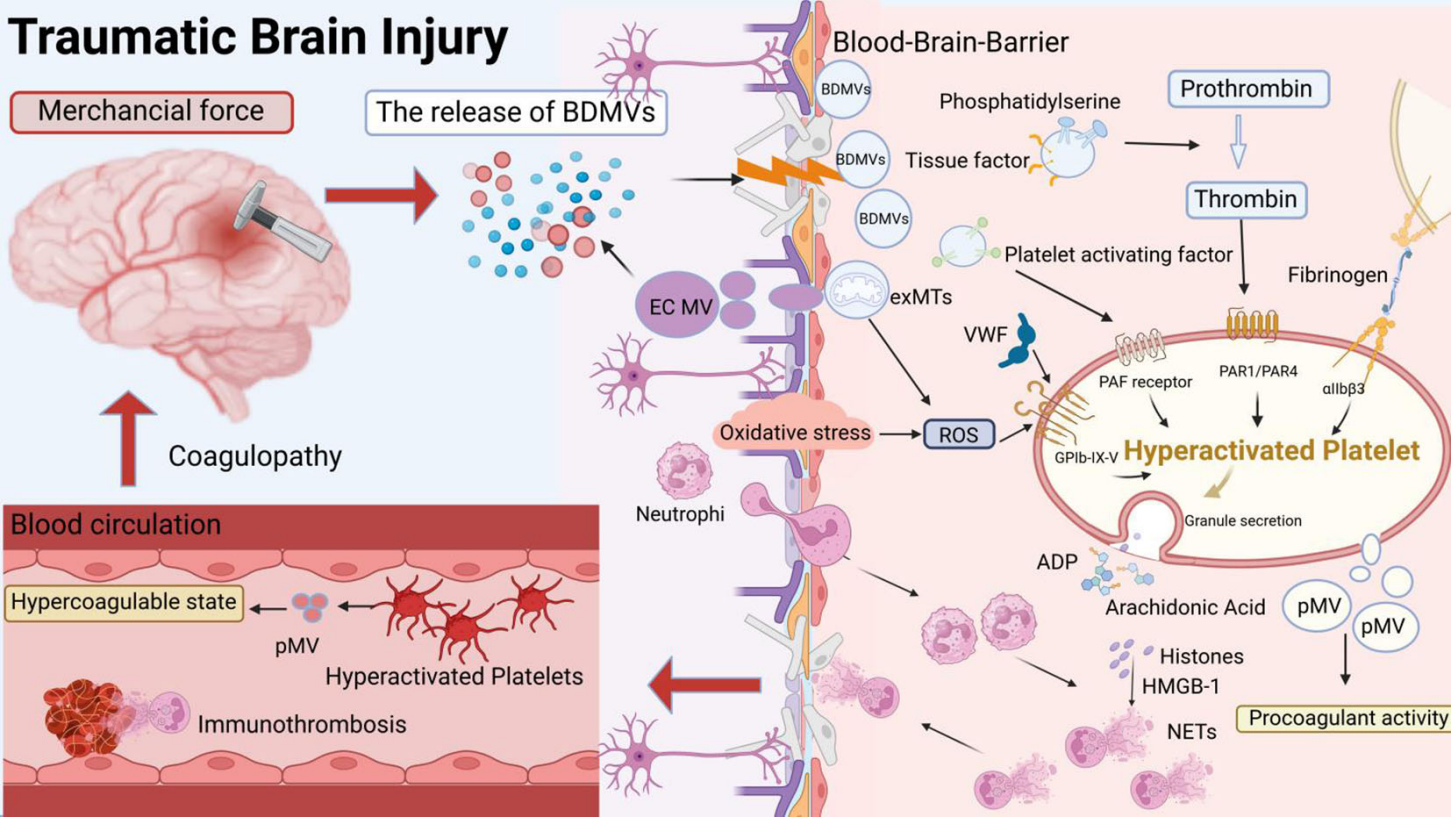
Mechanisms of platelet changes induced by traumatic brain injury. This figure shows how the disruption of blood brain barrier caused by traumatic brain injury affects platelets and results in changes in coagulation and inflammation. Traumatic mechanical forces cause brain-derived microvesicles (BDMVs) to be released into circulation after TBI. BDMVs have tissue factor, phosphatidylserine, platelet activating factor, exMTs, and other components which activate platelets through their binding to other receptors (GPIb-IX-V, PAR1/PAR4) and cause activation of platelets, secretion of granules from platelets, and external vesicle production. Blood brain barrier disruption also allows the migration of immune cells and pro-inflammatory mediators such as high-mobility group box 1 (HMGB-1) into circulation, where they interact with platelets, stimulate neutrophil extracellular trap (NETs) formation, and contribute to further neuroinflammation processes.

Moreover, HMGB-1 and histones promote the formation of neutrophil extracellular traps (NETs) via neutrophils (23, 50). The formation of NETs is triggered by various stimuli, including platelets, neutrophils, bacteria, microorganisms, and DAMPs, which is a protective process to prevent the spread of infection but also as a contributor to aggravate immune inflammation and cellular damage (6). Activation of platelets by HMGB-1 and histones promotes the formation of NETs via platelet-neutrophil interactions. Neutrophils release their nuclear components including DNA, histones, and neutrophil granule constituents, forming a web-like extracellular network to play an important role in coagulation and inflammation. The histones released by NETs, among which citrullinated H3 exerts proinflammatory and prothrombotic effects, while H4 is cytotoxic, thereby contributing to antimicrobial defense against infection (43, 46). These findings therefore represent a link between platelet function and the innate immune response, contributing to the development of inflammation and microvascular thrombosis after TBI. These data also provide insight into the complex interaction among HMGB-1, histones, platelet activation, NET formation, and immunomodulation. Thus, HMGB-1 and histones serve as critical mediators of platelet immunomodulation by releasing TLR4-dependent cytokines and promoting NET formation. Therefore, this data establishes a connection between platelets and the sterile inflammatory process associated with TBI (51, 52). **Figure 1** demonstrates the progression of platelet dysfunction after TBI.

#### 2.1.4 Platelet Biphasic effects on cerebral tissue

Some findings also indicate platelets exert biphasic effects on Cerebral Tissue. On the one hand, platelet can regulates TBI-induced injury and neuroinflammation. Platelets enter the brain parenchyma in response to severe injury, initiating neuroregeneration by releasing growth factors, such as platelet-derived growth factor (PDGF), neurotrophins, and anti-inflammatory mediators. In preclinical studies of traumatic brain injury, human platelet lysate has shown neuroprotective and neurorestorative properties, including reduced inflammatory cell numbers, decreased oxidative stress, and reduced synaptic loss (24, 53). For example, trauma-derived platelet releases ameliorates trauma-derived plasma-induced increases in endothelial permeability by regulating redox and antioxidant pathways (54). These mechanisms illustrate the significant contribution of platelet granule secretion to the regulation of immune-inflammatory crosstalk, leukocyte recruitment, BBB integrity, and neuroinflammation after TBI, by modulating injury progression and eventual recovery (55).Additionally, platelets express membrane receptors such as CLEC-2, which regulate inflammatory cytokine profiles and maintain the integrity of the blood-brain barrier. On the other hand, platelet activation can increase neuroinflammation by promoting microglial M1 polarization or disrupting the blood-brain barrier (5, 6, 56). Together, these mediators increase platelet activation and recruit and activate immune cells, leading to increased inflammation and angiogenesis in injured brain tissue. When platelets are activated or undergo death, they also shed pEVs from the plasma membrane, which contain a large number of proteins, receptors, lipids, and other components. Collagen and thrombin facilitate the production of platelet extracellular vesicles (10, 57). These vesicles possess both anticoagulant and procoagulant properties. Upon release, they can participate in various cellular processes such as hemostasis, inflammatory responses, and intercellular communication (54, 58, 59).This highlights the role of platelets on both sides of the injury continuum.

#### 2.1.5 TBI therapeutic targets

TBI induces a complex platelet dysfunction that oscillates between hyperreactivity and exhaustion, contributing to both hemorrhage and microvascular thrombosis. In clinical conditions, platelets are primarily used to assess changes in coagulation function after TBI. Evaluation of platelet function parameters and coagulation markers can provide a preliminary indication of whether a patient is in a hypercoagulable or hypocoagulable state (60), as well as whether early thrombotic events have occurred (61, 62). Nowadays, with the deep study of coagulation and inflammation, the therapeutic landscape outlined in **Table 1** reflects a paradigm shift from conventional hemostatic resuscitation toward targeted modulation of platelet signaling, endothelial integrity, and thromboinflammatory cascades.

**Table 1:** Potential therapeutic targets for traumatic brain injury-induced platelet dysfunction.

Drug/compound	Signaling pathway	Therapeutic roles	Blind spots and risks	Reference
Amitriptyline	Increase Sphingiosine-1-phosphate and ceramide levels	S1P modulates platelet aggregation affecting thrombosis, hemostasis, and wound healing	•Exact mechanism role of S1P in platelet aggregation is unknown •Anticholinergic reactions may affect consciousness assessment	(63–65)
Desmopressin	Exocytosis of vWf and VIII	Decrease antiplatelet-associated intracranial hemorrhage and enhance thrombogenesis	Its poor neurological outcomes need longer term functional outcome investigations	(66, 67)
Tranexamic Acid (TXA)	Binding to plasminogen and blocking the interaction of plasminogen with fibrin	Prevent/Reduce hemorrhage	It may produce higher rates of venous thromboembolism	(2, 68)
Aspirin	•Inhibit COX-1 pathway to inhibit the platelet • Express neuroprotective proteins	Inhibit platelet aggregation Have a neuroprotective role in improving the outcome and recovery	•The variability in aspirin effect of platelet aggregation in response to ADP •Uncontrolled bleeding	(63, 64, 69)
Clopidogrel	•Inhibit P2Y12 receptors to inhibit ADP-mediated platelet activation and aggregation • Express neuroprotective proteins	inhibit platelet activation and aggregation	•Increase intracerebral haemorrhage (ICH) risk •risk of mortality	(69)
Platelet transfusion	Reverse platelet inhibition	Normalize platelet counts and functions	Unable to independently examine the effects of plasma and platelets on outcomes	(70, 71)
A Cold-stored whole blood	Blood supply and Reverse platelet inhibition	Normalize platelet counts and functions	platelet function has not been evaluated in cold-stored WB transfusions.	(72)
Recombinant platelet CLEC-2	Reduce microglial activation and improve blood brain barrier integrity	Address neuroinflammation	The research was limited to animal studies, and the drug’s effects on humans still require further investigation.	(53)
Human platelet lysates (HPLs)	Produce a rich assortment of neurotrophic factors, antioxidants, and anti-inflammatory proteins	Improve neurorestorative effects	Clinical management requires to be evaluated	(73)
Mesenchymal stem cells (MSCs)	MSCs are actively mobilized to damaged tissues	Paracrine and immunomodulatory potential	The efficiency of the targeted migration of need further development	(74)

### 2.2 Platelet function alterations after traumatic hemorrhagic shock (THS)

Hemorrhagic shock is a leading cause of preventable death in trauma patients due to large volume of blood loss and a state of poor circulation (75, 76). Hemorrhagic shock often precipitates TIC, a complex hemostatic dysfunction that drives uncontrolled bleeding and serves as a primary determinant of adverse outcomes in trauma patients. In this context, platelets act as the primary link between hemostasis and innate immunity (77). Following an initial hypocoagulable state, a prothrombotic shift occurs 48 to 72 hours post-injury, resulting in increased risk for venous thromboembolism and multi-organ failure. After shock, platelets exhibit a transient phase of hyperreactivity, followed by functional exhaustion and a profound inability to aggregate, a dysfunction that complicates both resuscitation and thromboprophylaxis strategies. The lethal triad of coagulopathy, hypothermia and acidosis each contributes to worsening coagulopathy (78). Coagulation factor depletion and dilution due to trauma and volume resuscitation are major factors in the development of TIC, specifically fibrinogen and factors V and VIII; this results in decreased thrombin generation and impaired fibrin formation (79).

#### 2.2.1 Endothelial injury and rapid platelet activation after THS

During the early stages of THS, the endothelium undergoes rapid destruction as vascular beds sustain mechanical injury and ischemic hypoxia. This results in exposure of subendothelial collagen and the release of a large amount of tissue factor into the circulation. During this stage, endothelial cells also release large amounts of ultralarge vWF multimers, which bind to exposed collagen and platelets via their GPIbα and GPVI receptors, promoting firm attachment and rapid platelet binding to sites of injury (80). Damaged cells also release ADP, which activates P2Y1 and P2Y12 (81), and locally generated thrombin can activate PAR1/PAR4 on platelets, promoting calcium mobilization and “inside-out” activation of αIIbβ3 through both G-protein coupled receptor cascades and immunoreceptor tyrosine-based activation motif (ITAM)-linked signaling pathways (82).

THS induces the breakdown of endothelial glycocalyx to promote the release of heparan sulfate, which can prevent the adhesion of platelets to have an anticoagulant effect (83). In addition to this, inflammatory cytokines like IFN-γ can bind to heparan sulfate to inhibit its function through an *in vitro* test. From that, it is suggested that cytokines are not only pro-inflammatory but also alter endothelial integrity (84).

Platelets are further activated by the synthesis and release of thromboxane A2 (TXA2), which exerts its effects through the thromboxane prostanoid (TP) receptors. A recent study of TXA2 analogs revealed a complex relationship between TP and IP receptors. Further activation of TP receptors by TXA2 appears to lead to reversible platelet aggregation. This suggests that platelet activation pathways may have multiple temporal variations during the shock state (82, 85). Another example of how an exogenous factor might influence platelet response to the environment is nicotine. Nicotine can reportedly activate platelets via PAR4 and the PI3K/Akt pathway (86).

THS leads to cell death and stress, resulting in the large release of endogenous DAMPs, including ATP, HMGB1, histones, and S100 proteins, as well as inflammatory cytokines (IL-6, TNF-α). In addition, DAMPs interact with TLRs on platelets, initiating intracellular signaling that results in degranulation, P-selectin exposure and release of proinflammatory chemokines (87). This promotes a functional reprogramming of hemostatic aggregation into a mode of immunothrombosis, in which platelets actively help regulate and support neutrophils and endothelial cells in creating extracellular traps and promoting endothelial inflammation. Accordingly, platelet activation serves as a bridge between coagulation and innate immunity during critical illness (88).

#### 2.2.2 Platelet contribution to hemostasis after THS

THS creates an environment characterized by high levels of concurrent stimulation and low levels of inhibition in platelets (89), leading to significant dysfunction of major platelet activation pathways (1, 90, 91). Injury to endothelial cells, the presence of tissue factor and high levels of thrombin production (92), and subsequent release of ADP from the lysed cells all contribute to strong stimulation of platelet receptors, including the protease-activated receptors (PAR) (93). The early phase of thrombin formation shows strong stimulation of both PAR1 and PAR4, together with an initial maximum platelet response. However, as shock persists and ongoing levels of agonists (thrombin, ADP) are circulating, there will be an adaptive downregulation of surface receptors on platelets due to continued stimulation of both PAR1 and PAR4, for which CD93 will negatively influence the rate of internalisation of PAR4 (92–94).

During trauma hemorrhage, platelets transform into procoagulant balloons and begin to produce microparticles, which is induced by histone H4 (95). As a result of tissue damage and shock, histones are released and interact with circulating platelets through their direct actions on platelet membranes. Once activated, platelets undergo morphological changes and release granules; α-granules contain most of the major coagulation factors, such as vWf and fibrinogen, while dense granules contain smaller molecules, such as ADP and serotonin, that play a critical role in the normal processes of hemostasis and thrombosis (89, 96). In addition, as a platelet activator, ADP serves as an amplifying signal upon excretion, resulting in the recruitment of other circulating platelets for clustering and/or morphological changes. While platelet activation continues, the granule excretion machinery remains operational, providing an ongoing response capability (97). Thus, the ongoing availability of granule excretion machinery will provide a critical storage of factors necessary for maintaining residual hemostatic capability, especially while the body is under pathological stress, when primary platelet activation systems may be impaired (80, 98).

During massive hemorrhage following vascular injury, the hemostatic function of platelets is severely compromised. Nonetheless, their participation remains necessary to establish initial hemostasis. In the context of vascular injury, platelets can adhere to exposed subendothelial collagen and vWF by rapidly recruiting platelet glycoprotein Ib-IX-V and integrin α2β1 to the site of injury, a process termed adhesion. Once platelets are activated, they release ADP and serotonin from dense granules and produce TXA_2_ via cyclooxygenase-1. Besides, platelets undergo significant cytoskeletal reorganization by extending filopodia and lamellipodia to maximize the surface area of the exposed extracellular matrix (85). This change of shape is vital in helping to stabilize platelet adhesion under circumstances of compromised hemodynamic forces and persistent bleeding, such as are seen during shock, because hemodynamic forces have been damaged due to low-flow, low-shear states.

Ultimately, stable platelet aggregation occurs through conformational activation of integrin αIIbβ3, which leads to high-affinity binding of platelets to fibrinogen and vWF (77). Platelet crosslinking enables firm consolidation of the hemostatic plug at the site of endothelial injury. Although the hemostatic aggregate caused by consumption of coagulation factors/excessive bleeding during hemorrhagic shock will compromise the durability of the thrombus, the ability of platelets to aggregate through integrin-mediated means and secrete their granules after initial aggregation assures that there is residual thrombogenic potential for a critical time window for resuscitation and/or surgical hemostasis (97, 98).

#### 2.2.3 Sustained hypoperfusion consumes platelets

Hypoperfusion leads to depletion of coagulation factors and inadequate thrombin generation. This affects the ligands required for the PAR pathway, as they require continuous stimulation to maintain activity. As trauma-induced hyperfibrinolysis progresses, there is a decrease in both thrombin and coagulation factor concentrations after an earlier increase in thrombin. The failure to produce sufficient thrombin prevents PAR activation, further worsening platelet function and the body’s ability to stop bleeding (96). Prolonged exposure to higher concentrations of agonists also leads to receptor desensitization and internalization especially poor responses to ADP and AA (29); The reason for such phenomenon cannot be fully examined both in TBI and THS, but it seems to occur due to a lack of plasma thrombin supporting the PAR pathway and is independent of hypoperfusion and platelet count. This state of platelet dysfunction/insensitivity is referred to as “platelet exhaustion” or “shock-induced hyporeactivity”. Through mechanistic studies, the relationship between CD93, surface expression, and PAR4 activation has been established (99); thus, the absence of CD93 accelerates both PAR4 internalization and desensitization (95). In repeated stimulation protocols, PAR-mediated responses are found to diminish more rapidly than GPVI-mediated responses, indicating distinct kinetics for both receptors with respect to priming and desensitization (100).

Tissue injury causes a loss of both coagulation factors and platelets from the hypoperfused area. Hypoperfusion leads to lactic acidosis and tPA release, which increases hyperfibrinolysis and disrupts fibrin clots and platelet plugs. In addition, hypoperfusion and acidosis in hemorrhagic shock produce large amounts of thrombin and tPA, leading to systemic consumptive coagulopathy. This causes elevated fibrin degradation products and D-dimer levels, as well as altering GPVI signaling and integrin function, thereby profoundly impacting platelet aggregation and activation (101). Initially, platelet and endothelial cell activation promotes hemostasis but with continued hypoperfusion, there is the potential for consumptive thrombocytopenia through ongoing activation, clearance of platelets and depletion of ATP (102). Moreover, tissue ischemia caused by hypoperfusion can subsequently activate endothelial cells to increase their expression of thrombomodulin, which will facilitate activation of protein C. Activated protein C has two functions: First, it inhibits the actions of factor Va and factor VIIIa, leading to continued consumption of coagulation factors and ultimately further impairing the coagulation cascade (75).

At this stage of the disease, defects in platelet aggregation have been shown to display both an energy-dependent characteristic and a signal-dependent characteristic; for example, a lack of ATP disrupts the outside-in signaling pathway of the integrin αIIbβ3 (103), which ultimately reduces the activation of PKC, leading to the formation of clots that are weak and likely to disintegrate early on. Both clinical and research studies on trauma patients’ primary blood platelets show consistently lower platelet aggregation in platelet-rich plasma compared to healthy people. This is likely due to dysfunction of their receptors, which likely results in diminished intracellular signaling pathways, including reduced calcium mobilization and reduced PKC activation - two important mechanisms necessary for platelet activation (104). PKC isoforms are essential for platelet life. If there are not enough isoforms of PKC, platelets will become more reactive to their environment and have a shorter lifespan. PKC, along with integrins, helps stabilize clot formation and control bleeding after trauma when either signaling pathway does not function properly or in a timely manner (105). With platelet function and blood clotting being compromised by this breakdown of signaling and integrin pathways, it is extremely important to maintain platelet function and signaling during times of significant blood loss in order to obtain effective clotting.

#### 2.2.4 Acidosis promotes platelet dysfunction

There is scientific literature showing that hypoperfusion and shock cause acidosis, which therefore produces less thrombin, causing platelet dysfunction and contributing to worsening bleeding outcomes (78, 106). In turn, metabolic acidosis is part of THS and is caused by tissue hypoperfusion and anaerobic metabolism. This process decreases the intracellular pH and significantly disrupts key enzyme activity in platelets. The two major pathways affected are the cyclooxygenase-1 (COX-1) pathway and thromboxane A2 (TXA2) synthesis, both of which are critical for platelet activation and platelet aggregation (85).

When platelets experience acidosis (pH below 7.2), their ability to adhere to fibrinogen is reduced, as integrin αIIbβ3 becomes inactive. In this context, studies have demonstrated that acidosis impairs platelets’ ability to form stable aggregates. Acidosis has also been shown to induce greater platelet apoptosis and increased phosphatidylserine exposure on platelet surfaces, indicating an increase in platelets undergoing programmed cell death (88). There is also a considerable reduction in the function of platelet receptors, such as glycoprotein Ibα (GPIbα), in patients suffering from THS, because the shedding of GPIbα by metalloproteinases such as ADAM17 and the oxidative damage from ROS decrease the amount of GPIbα that can adhere to platelets and participate in thrombus formation (107, 108). As platelets undergo activation, their extracellular receptors like GPIb are shed from the surface of the platelet, while GPVI and integrin αIIbβ3 are preferentially upregulated to provide the larger platelets with the ability to adhere more effectively (108, 109). Although the aggregation of platelet is induced, acidosis can still enhance granule secretion to express more P-selectin to bind to neutrophils and form platelet-neutrophil complex during THS. An *in vitro* experiment suggested that the formation of platelet-neutrophil complex indicate the pro-inflammatory capacity of platelet (110).

Acidosis can also activate the complement system, which is part of the innate immune response and shares similarities with coagulation systems (111). Complement not only bridges innate and adaptive immunity, but also interacts with platelets and the coagulation system (112, 113). Under physiological conditions, platelets are closely linked to the complement system through complement receptors and complement regulatory factors expressed on their surface (114). The complement system is massively activated when pH drops below 7 and can recruit immune cells and platelets mediate these cells’ function through platelet components(CD40, P-selectin, CD154)and factors, such as platelet factor 4 (PF4, CXCL4), thus greatly underscores the role of platelets in amplifying immune inflammation and the complexity under acidotic conditions (115). Although the specific mechanisms underlying the interaction between platelets and the complement system are still unknown, many studies indicate that complement C3b promotes, or at least participates in, platelet activation and stimulation during platelet–bacteria interactions (116, 117). What is certain is that the cross-talk between the complement cascade and platelets occurs on the platelet surface and represents a pathological reaction in which P-selectin released by platelets acts as a receptor (117, 118). In addition, the cytolytic effect of the membrane attack complex promotes ADP release, thereby facilitating platelet aggregation (119). As the key participant in thromboinflammation, platelets activated by complement activation products tightly link these two systems together in disease progress.

Moreover, throughout the resuscitation process, platelet dysfunction is compounded by the oxidative stress produced as a result of ischemia-reperfusion injury due to resuscitation efforts. This oxidative stress alters the function of membrane-bound lipids and proteins that are integral to platelet adhesion and aggregation. Furthermore, oxidative modifications to platelets create a pro-inflammatory phenotype, as they increase the release of pro-inflammatory cytokines and chemokines (120).

#### 2.2.5 Hypothermia blunts residual platelet function

Hypothermia is a common condition in trauma patients. It can aggravate hemorrhagic shock and platelet dysfunction (121). When a person’s core body temperature drops below 35 °C, their ability to form blood clots is compromised given that hypothermia impairs platelet function and the enzymes responsible for blood coagulation (122). As body temperature decreases, the catalytic function of enzymes involved in hemostasis is attenuated, delaying clot formation. Specifically, hypothermia affects enzymes that produce thromboxane and those that produce certain granules, which are required for platelet activation and aggregation (123). THS compromises platelet granule release, thereby diminishing the positive feedback signals required for effective platelet activation and aggregation. When the release of dense granules is impaired, ADP becomes less available for platelet activation and aggregation. When the release of alpha granules is impaired, less vascular endothelial growth factor (VEGF) and PF4 are released, potentially delaying vascular healing and tissue repair after injury (124, 125).

**Figure 2:**
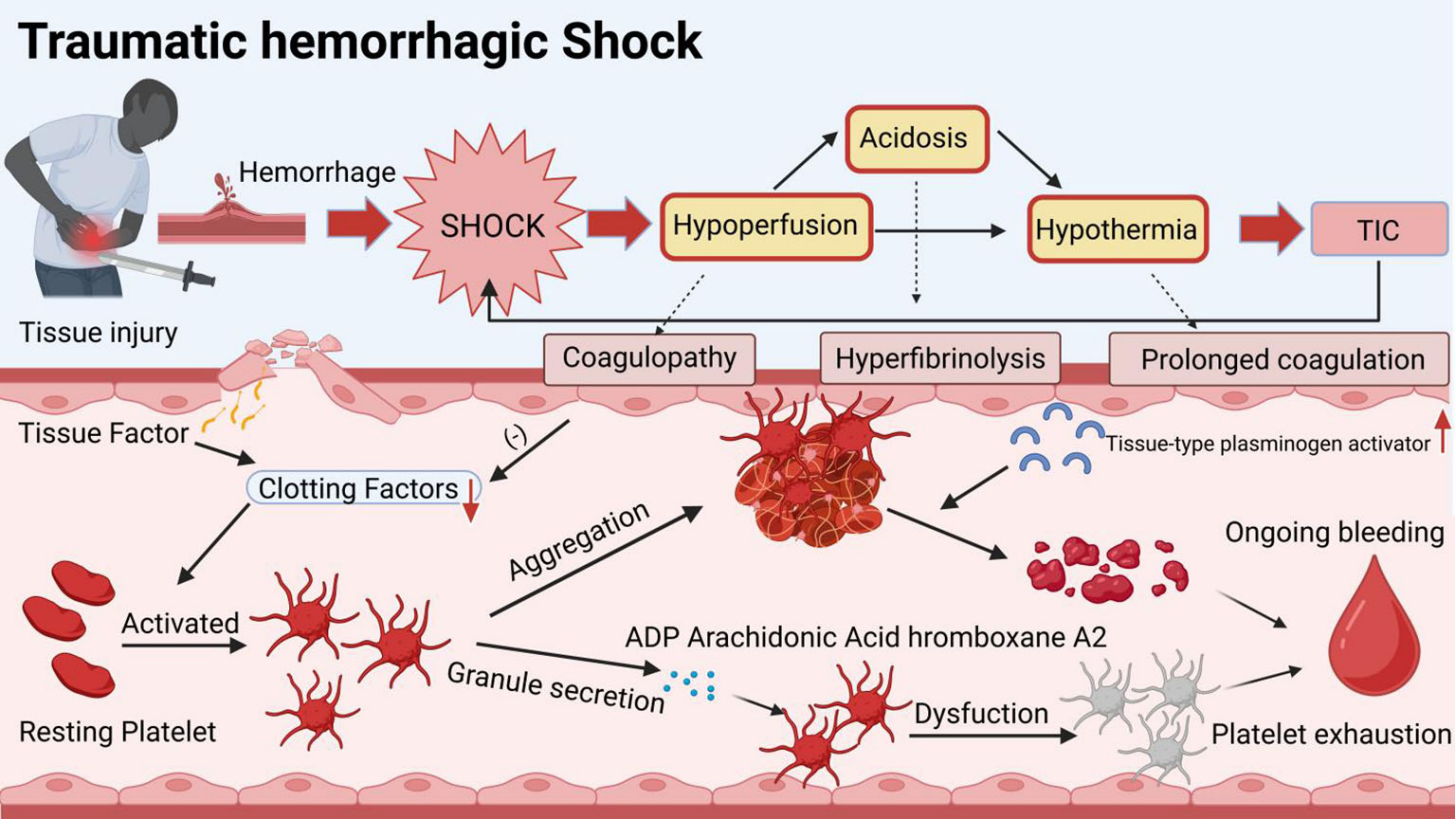
Mechanisms of platelet changes induced by Traumatic hemorrhagic shock. This figure presents the reciprocal and cyclic nature of the interrelationship between trauma, shock, and coagulopathy. The three factors create a sequential and complementary network of platelet injury through the mechanism of hypoperfusion → acidosis → hypothermia. On the one hand, there is the metabolic crisis initiated by hypoperfusion and the disruption of barrier function; on the other hand, acidosis has a negative effect on the function of critical enzymes and receptors involved in coagulation and also increases the rate of hyperfibrinolysis; and, finally, due to the effect of hypothermia, the kinetics of secretion and reactions are slowed. Together, these three factors produce platelet exhaustion by means of four main mechanisms: signal blockade, receptor dysregulation, impaired aggregation, and secretion defects. These processes represent the basic cyto-pathological mechanism for trauma-induced coagulopathy(TIC).

Many studies have reported that hypothermia prolongs clotting times, slows clot growth, and reduces platelet agglutination in response to ADP, arachidonic acid, and the thrombin receptor. Rewarming after being hypothermic can create an increased risk of developing blood clots due to an increase in platelet synthesis and activation (126, 127). **Figure 2** demonstrates the progression of platelet dysfunction after THS.

#### 2.2.6 THS therapeutic strategies

To date, blood transfusion remains a critical intervention in the management of THS. From the perspective of platelets, platelet count provides a direct reflection of hemostatic function. In addition, researchers have demonstrated that prehospital plasma transfusion can correct acidosis, hypothermia, and coagulopathy (128). However, whether the leukocytes in transfused blood components influence patient outcomes remains a matter of debate (129). As shown in **Table 2**, platelet dysfunction due to traumatic hemorrhagic shock is a major pathological component of TIC driven by complex mechanisms and disruption of multiple signaling pathways. Future research should focus on defining the optimal timing for different therapeutic modalities in relation to the various phases of traumatic hemorrhage and examining combinations of therapy that will go beyond just correcting platelet dysfunction and attempt to restore systemic hemostatic homeostasis.

**Table 2:** Potential therapeutic targets for traumatic hemorrhagic shock-induced platelet dysfunction.

Drug/compound	Signaling pathway	Therapeutic roles	Blind spots and risks	Reference
Tranexamic acid, fibrinogen concentrate, and prothrombin complex	Inhibit fibrinolysis and promote coagulation.	Promote hemostatis	Influence on coagulation parameters and specific thrombotic complications require further exploration.	(130)
Cathelicidin-HG	Targeting GPVI and blocking PI3K/AKT/ERK, RAP1	Reduce microthrombosis	The influence of normal coagulation function remains unclear	(131)
Synthetic platelets	Bypass storage limitations of native platelets	Reduce blood loss and restore hemostasis	•lack human trial data •Its function and efficiency compared with natural platelet remain unknown.	(132)
Wnt agonist iCRT-14	Recover Wnt/β-catenin cellular pathways	Reduce apoptosis	•Clinical data are needed to support Pleiotropic Mechanisms of Wnt/β-catenin and NFκB signaling pathways.	(133)
Sibanin	Inhibit P2Y12 and GPVI receptors	Reduce thrombus formation	Its benefits are limited to the DIC stage	(134)
Modified Whole Blood	Supply whole blood	Improve platelet function	further studies on thrombin synthesis and coagulation are needed	(135)
Blood product transfusion (1:1:1 FFP:platelets:PRBCs)	Supply blood cells, plasma and platelets to improve hemostasis	Blood volume replenishment	The optimal transfusion ratio still requires further investigation	(136)
The pEVs preparation	Mitigate the development of ischemia and metabolic acidosis	Provide hemostasis	•Donor variability and potential undesired cellular components may affect its efficacy •Whether it can correct platelet dysfunction needs further study	(137, 138)
Platelet-rich plasma (PRP) applications	Release platelet growth factors	Wound healing	•Viable preparation and application methods influence its clinical outcomes •Whether it can correct platelet dysfunction needs further study	(139, 140)
miR-19b oligo inhibitor	Restoration of endothelial function and permeability	Reduce post-shock lung injury, inflammation and hyperpermeability.	•Molecular mechanism underlying the mRNA reduction is not clear •Whether miR-19b promotes the known pro-inflammatory cytokine release from endothelial cells following hemorrhagic shock remains unknown.	(141)
Mesenchymal stem cells (MSCs)	Decreased proinflammatory cytokines	Enhance bacterial clearance	The efficiency of the targeted migration of need further development	(142)

### 2.3 Platelet function alterations after severe burns

Burn injury is a unique, complex trauma leading to widespread systemic dysfunction such as bleeding and coagulopathy. Although there are some similarities between burn injury-induced coagulopathy and acute traumatic coagulopathy, the underlying pathophysiology of these two types of coagulopathy involves significant and systemic endothelial activation, continued systemic inflammation/dysfunction that leads to severe exogenous activation of inflammation, and a prolonged hypermetabolic state (12). Mechanically traumatic injuries primarily trigger an early coagulopathic response via tissue factor release and hematologic shock; however, burn injuries result in an ongoing interaction among damaged endothelium, circulating platelets, and innate immune cells, leading to a dynamic, biphasic platelet phenotype (143).

Burn-induced platelet dysfunction refers to more than decreased ability to stop bleeding; it also affects leukocyte recruitment, vascular permeability, and tissue repair. Conversely, the release of growth factors from platelet α-granules, such as PDGF, TGF-β, and VEGF, triggered by activation of the coagulation cascade, is a major contributor to angiogenesis and epithelial regeneration. However, although these factors can facilitate healing, excessive or dysregulated platelet activation may ultimately impair the reparative process (144).

#### 2.3.1 Platelet hyperreactivity and early hemoconcentration

When someone has a severe burn, their body will respond within 24 hours with many changes in how platelets function. One major change is that when fluid from capillaries leaks out into the surrounding tissue and causes swelling as known as edema, the volume in blood vessels decreases and blood cells become more concentrated (144, 145). A decrease in plasma volume in the blood vessel can make the number of platelets appear increased due to the concentration effect, but this can be confusing because it may reflect an actual decrease in platelet number, known as “consumed” platelets. During the formation of fibrin and the aggregation of platelets, other coagulation factors and platelets are becoming consumed, which will lead to the development of a consumptive coagulopathy; whereas due to the severity of the coagulopathy, a person’s platelet count may look normal or elevated, because hemoconcentration has masked the effect of consumption (12).

Platelets become more active during stressful situations due to the effects of stress hormones. Stress hormones, such as elevated circulating catecholamines, pro-inflammatory cytokines (such as IL-6), and thrombin production, influence platelet activity (146). Studies have demonstrated that platelets collected at this time exhibit higher levels of aggregation when exposed to agonists such as collagen or ADP, produce more thromboxane B2, and demonstrate greater platelet activation by expressing more of the activation markers CD62P (P-selectin) and activated GPIIb/IIIa (147). While hyperreactive platelets are initially a compensatory response, they also increase the risk of thrombus formation in the microcirculation, thereby prolonging ischemia in tissues (148).

Platelet counts and functions change after burns across multiple animal models, with evidence of a hypercoagulative state and enhanced platelet reactivity and thrombin generation persisting for about 1 week following burns in mouse studies (149). Laboratory cats sustained thermal injury and smoke inhalation, and the researchers also found evidence of pre- and hyperactivation of platelets. There was an increase in the number of pEVs, as well as an increase in the expression of P-selectin, a surface molecule found on platelets (146). In a sheep study looking at the changes in coagulation and platelets after burns over time, it was shown that immediately after a burn, its hypercoagulation was present. After that point, it was hypocoagulated because of decreasing platelets and consumption of coagulation factors. Finally, during the recovery phase, the sheep had a hypercoagulated state because of the increased number of platelets (150).

#### 2.3.2 pEV release after burns

The morphological change associated with the hyperacute state of cell death was platelet ballooning. Therefore, the morphology of ballooning platelets demonstrates an activation phenotype (95, 146), characterized by increased pseudopod formation, leading to platelet spheroid enlargement. Although ballooning platelets do not aggregate, they act as a paradoxical stimulant for the shedding of pEVs. These submicron-sized particles derived from the platelet membrane have been identified as a major contributor to burn coagulopathy. Through proteomic analysis of pEVs obtained from burn patients, there are 268 differentially expressed proteins (191 increased and 77 decreased) in burn patients compared to healthy pEVs (151). In addition to their procoagulant activity due to surface-exposed phosphatidylserine and tissue factor, the unique cargo of pEVs indicates that they are coagulation mediators in systemic communication with distant cells (152). Thus, pEV shedding early after injury promotes and maintains a prothrombotic state following the hyperacute phase of injury.

The analysis of pEVs indicated quantitative and qualitative differences after burn injury from early post-burn time points to late post-burn time points (151). The pEVs may have reduced counts, however the procoagulant activity per vesicle remains high and the proteomic composition of pEVs is now skewed towards complement activation proteins, acute phase response proteins, and proteins associated with cell death pathways. Thus, pEVs may play a role in the initiation of burn coagulopathy as well as being an ever-changing biomarker indicating disease progression and prognosis (151, 152).

#### 2.3.3 Multifactorial Thrombocytopenia and consumptive coagulopathy

Thrombocytopenia typically appears within 48–72 hours after a burn and is due to multiple factors with prognostic value (153, 154). First, continued platelet consumption occurs at the wound site and within the systemic microvasculature, driven by the ongoing formation of disseminated microthrombi from circulating platelets. Second, platelets become sequestered in injured organs, such as the liver, spleen, and pulmonary vasculature, given that activated platelets adhere to the inflamed endothelium of these organs (12). Third, immune-mediated platelet destruction occurs due to autoimmune responses and complement activation triggered by the burn injury, leading to accelerated platelet clearance (154).

Aggressive crystalloid administration during resuscitation will reverse hemoconcentration through hemodilution, thereby revealing the underlying thrombocytopenic deficit. Thrombocytopenia severity correlates directly with total body surface area burned and independently predicts mortality, highlighting the value of serial platelet counts as a prognostic tool. In addition, the burned tissue fosters consumptive coagulopathy via endothelial disruption, exposing collagen and tissue factor to the blood, thereby activating the coagulation process and leading to fibrin formation and platelet aggregation at the site of injury (12, 13).

#### 2.3.4 Platelet count recovery

During the second week after suffering an injury from severe burns, patients who survive usually will have their platelet counts and functions returned to normal levels due to the production of new platelets (megakaryocytes) stimulated by thrombopoietin. Increased numbers of newly produced platelets are released into circulation as a result of the newly produced and released platelets being more sensitive to ADP and arachidonic acid as they have been primed by inflammatory mediators (147, 155). So while the functional rebound of platelets restores the ability to perform hemostasis, it causes a prothrombotic state which may create the potential for thromboembolic complications if not managed properly.

Immunohistochemical studies of burn areas removed during this time typically show significantly increased levels of procoagulant factors in microvessels, such as TF and factor XII, with large numbers of platelet-rich clots forming in the dermal microvasculature. These microthrombi not only deplete platelets and coagulation factors but also critically disrupt the delivery of oxygen and nutrients to surviving tissue, increasing the risk of progression from partial-thickness injury to full-thickness necrosis. As a result, platelet-related microvascular occlusion directly obstructs wound healing and increases the total amount of tissue lost (156, 157).

#### 2.3.5 Platelets as immune mediator

After a burn injury, inflammatory response can lead to serious systemic consequence (158). Platelets serve as mediators of both immunity and coagulation throughout the entire healing process. Immediately following burn injury, platelets form microaggregates upon contacting the endothelium. These microaggregates are essential for the formation of early microthrombi within the microcirculation as part of the host-protective immune response (159). By sequestering pathogens, they help prevent their dissemination and limit further tissue damage. Following severe burns, however, the process of immunothrombosis becomes dysregulated and leads to systemic microvascular occlusion and poor distribution of blood into the organs (160).

Platelets use surface molecules, such as CD40L and P-selectin, to trigger inflammation (160, 161). When activated, platelets express CD40L, which binds to CD40 on endothelial cells, leading to increased expression of adhesion molecules and the secretion of chemokines that recruit leukocytes to damaged areas and remote sites in the body (161). While this process is essential for protection against wound infections, it also perpetuates systemic inflammation and impairs endothelial barrier function. IL-1 released by platelets also increases vascular permeability, allowing leukocytes to move from blood vessels into tissue and continuing the inflammatory response (145, 162). Circulating monocyte-platelet aggregates indicate persistent platelet activation and predict burn severity and clinical outcomes (163).

Burn injuries cause DAMPs to be released in large amounts from necrotic tissue (158, 163). When these endogenous danger signals bind to platelet TLRs, especially TLR4, platelet activation occurs along with sterile inflammatory responses. The interaction between HMGB1 and TLR4 on platelets leads to granule release, integrin activation, and pEV release therefore enhancing coagulation processes and inflammation. This sterile inflammatory cascade will cause a patient to develop SIRS and if severe enough will lead to the development of MODS. Circulating DAMPs will be present and will cause persistent platelet activation throughout the post-burn period therefore relating the amount of tissue necrosis to the amount and duration of platelet mediated systemic injury (155).

#### 2.3.6 Sepsis and NETs

Severe burns will disrupt immune system and cause sepsis, a critical risk to burn victims (164, 165). Researchers find that flame burned patients have a higher risk for sepsis than scald burned patients to develop infectious complications (166). Due to sepsis, an individual’s immune system becomes primed for further infections and clotting, thereby causing additional injury after a burn (167). With sepsis, the innate immune system is activated. The immune system is activated by PAMPs from bacteria, by direct interaction of bacterial cell wall components with TLRs on platelet surfaces, and by platelet secretion of proinflammatory cytokines. Once activated, platelets promote NET formation through interactions between P-selectin on platelets and PSGL-1 on neutrophils, and they also promote the release of chemokines (168, 169). NETs are web-like structures composed of an extracellular DNA matrix that contains histones and proteolytic enzymes, which trap and kill circulating pathogens while also providing a structural scaffold for platelet adhesion and thrombin generation (168). Histones and DNA are also released from NETs, acting as DAMPs and further stimulating platelets and the coagulation cascade, creating a self-amplifying loop and consuming the platelets (170).

**Figure 3:**
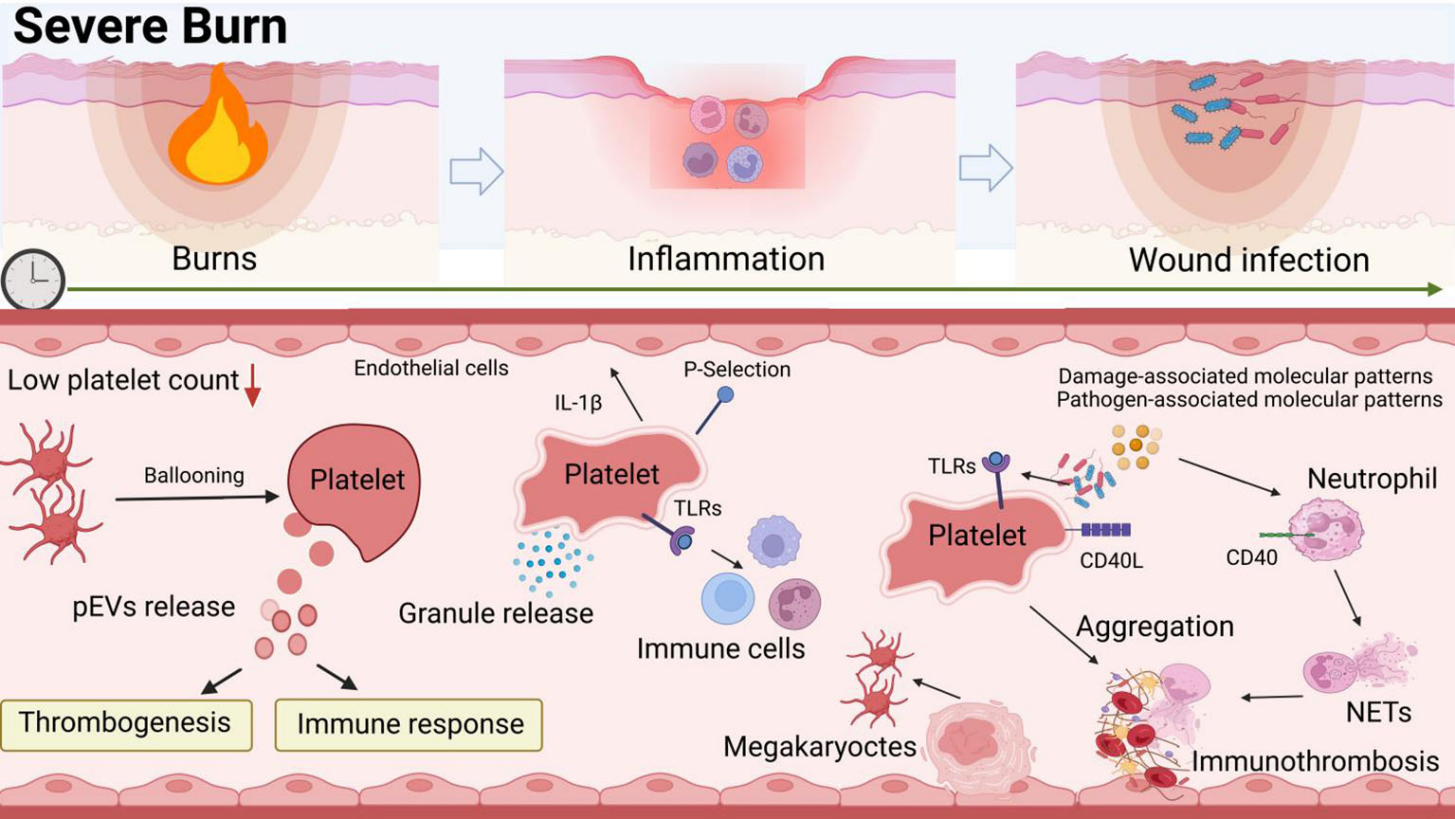
Mechanisms of platelet changes induced by severe burns. This figure illustrates how severe burns impact platelet function to ultimately lead to immunothrombosis. During the post-burn phase, platelets will exhibit reduced platelet counts and ballooning of platelets, as well as an increase in the release of platelet-derived extracellular vesicles (pEVs). In addition to the already established role of platelets in coagulation, after a burn, platelets are primarily involved in signaling related to inflammation. This relationship primarily relies on changes in platelet phenotype, recruitment of inflammatory cells, and coupling of coagulation with immune responses via the action of neutrophil extracellular trap (NETs).

Burn injuries and sepsis both compromise the endothelial glycocalyx, exposing surface receptors that recruit platelets and neutrophils. This integrates the thrombotic and inflammatory responses that usually occur at the endothelium. At the same time, megakaryocytes produce platelets rich in pro-inflammatory mediators and DAMPs in response to sustained inflammatory signals from burn injury or sepsis, thereby perpetuating the cycle of immune activation and dysregulation of coagulation (169). In addition, while NETs help contain pathogens from either a burn or an infection, the overproduction of NETs during sepsis caused by a burn impairs blood flow through the microcirculation and worsens tissue hypoxia, leading to progressive organ failure (169, 170). **Figure 3** demonstrates the progression of platelet dysfunction after severe burns.

#### 2.3.7 Therapeutic strategies

Platelet dysfunction caused by burns is often overlooked; however, it very much plays an important role in the coagulopathy and systemic inflammatory response following severe burns. In burn injuries, there is a chronic, two-phase process of platelet dysfunction compared to traumatic hemorrhagic shock, where there is mainly acute consumption of platelets and dilutional coagulopathy. In summary, the management of burn-induced platelet dysfunction is evolving from the nonspecific use of transfusions to mechanism-based, targeted therapies, as shown in **Table 3**. Future research will address the need for translatable research, identification of unique platelet-signaling signatures for burn injuries, and development of individualized algorithms for managing patients at risk or with both hemorrhage and thromboinflammatory complications.

**Table 3:** Potential therapeutic targets for burns-induced platelet dysfunction.

Drug/compound	Signaling pathway	Therapeutic roles	Blind spots and risks	Reference
Platelet-rich plasma (PRP)	Activated platelets release growth factors and other proteins through granule secretion	Promote cellular proliferation and differentiation to accelerate wound healing	• Combination therapies with other treatment modalities may affect efficacy • PRP preparation and administration are Lack of standardization	(171, 172)
Allogeneic cord blood platelet gel	A concentrate of platelets and growth factor	Modulate local inflammation and angiogenesis	Additional bioactive components from platelet sources need clinical assessment	(173, 174)
Human platelet lysate-loaded collagen-poloxamer foam dressing	Released epidermal growth factor (EGF) and platelet-derived growth factor (PDGF)	Promote wound healing and modulate platelet function	The management of stability, sustained release, and ease of application need further study	(175)
Topical heparin	inhibit inflammatory mediators, and improved local microcirculation	decrease local edema and pain perception	Require Standardized protocols and larger sample capacity	(176)
Pentoxifylline	Reduce platelet aggregation	reduce risk for vascular events	long‐term oral drug administration remains unknown	(159, 177)
Ulinastatin	Inhibit secretion of β-TG and PF-4 from platelet inhibits the release of inflammatory mediators from neutrophils	Improve microcirculation and inflammation	Longer term outcomes requires to be evaluated	(178, 179)
Xuebijing (traditional Chinese medicine)	Reduce platelet-derived growth factor and platelet activating factor levels	Improve platelet rheology	Xuebijing can not directly inhibit and kill pathogenic microorganisms	(180–182)
Thrombin-anchored bacterial cellulose (T-BC) dressing	Facilitates stable thrombin	Enable rapid hemostasis	Clinical management requires to be evaluated	(183)

## 3 Discussion

Researchers are learning how trauma, such as traumatic brain injury, hemorrhagic shock, and burns, impact platelets in respect to the hemostatic and immune roles of the platelets following injury. Regardless of what constitutes trauma, all such traumas have a common mechanism via the process of endothelial injury and DAMPs, which activates a series of processes involved in coagulation, fibrinolysis, and inflammatory response (184, 185). Lastly, the similarities among platelets associated with these types of traumatic conditions demonstrate similarities in their physiologic functionality (1, 77).

Recent research on platelets has established that they are more than just hemostatic cells; they are now considered integral partners in the pathophysiology of trauma (186). Their activation and functional changes not only assist in the management of trauma but can also accelerate disease progression through inflammatory pathways (187, 188). In this review of three types of trauma, we see both similarities and differences regarding the mechanism of action; nonetheless, each trauma type has a unique pathological mechanism of action and therefore modulates the function of platelets through unique pathways - a topic that has not been previously studied. These findings indicate directions for future research. Coagulation dysfunction caused by different trauma mechanisms is particularly problematic for standard interventions, leading to a need for individualized approaches that target specific pathophysiological mechanisms (189).

Overall, this review illustrates that platelets can serve both as protective agents and as potential contributors to pathophysiology following trauma. By recognizing and appreciating the complexity and variations in platelet responses to TBI, hemorrhagic shock, and burns, the field can more effectively implement precision medicine approaches that balance coagulation and immune modulation. This balanced view will not only help increase understanding of the mechanisms involved but also facilitate the development of novel therapies that may improve mortality and morbidity in critically injured patients.
